# Integrated Approach to Interaction Studies of Pyrene Derivatives with Bovine Serum Albumin: Insights from Theory and Experiment

**DOI:** 10.1021/acs.jpcb.2c00778

**Published:** 2022-05-18

**Authors:** Selvaraj Sengottiyan, Kakoli Malakar, Arunkumar Kathiravan, Marappan Velusamy, Alicja Mikolajczyk, Tomasz Puzyn

**Affiliations:** † Laboratory of Environmental Chemoinformatics, Faculty of Chemistry, University of Gdansk, Wita Stwosza 63, Gdansk, 80-308 Poland; ‡ Department of Chemistry, North Eastern Hill University, Shillong 793 022, Meghalaya, India; § Department of Chemistry, Vel Tech Rangarajan Dr. Sagunthala R & D Institute of Science and Technology, Avadi, Chennai 600 062, Tamil Nadu, India; ⊥ QSAR Lab Ltd., ul. Trzy Lipy 3, Gdansk, 80-266 Poland

## Abstract

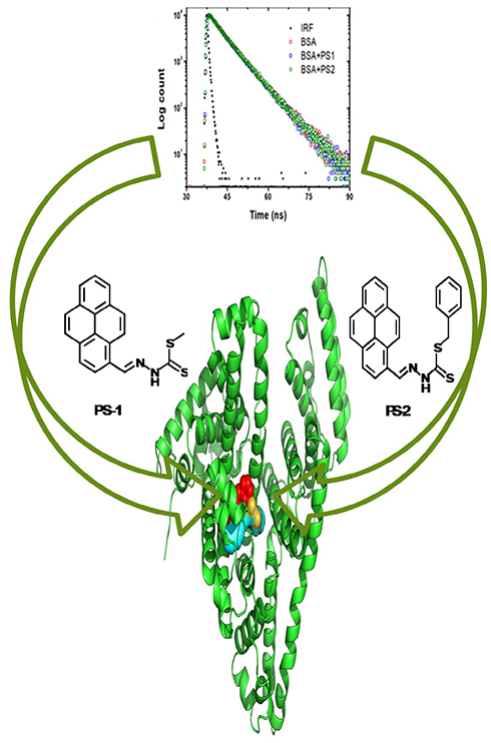

This work aimed to investigate the interaction of bovine serum albumin with newly synthesized potent new pyrene derivatives (PS1 and PS2), which might prove useful to have a better antibacterial character as found for similar compounds in the previous report [Low et al. Bioconjugate Chemistry2014, 12, 2269−228410.1021/bc500490725382115]. However, to date, binding studies with plasma protein are still unknown. Steady-state fluorescence spectroscopy and lifetime fluorescence studies show that the static interaction binding mode and binding constants of PS1 and PS2 are 7.39 and 7.81 [*K*_b_ × 10^5^ (M^–1^)], respectively. The experimental results suggest that hydrophobic forces play a crucial role in interacting pyrene derivatives with BSA protein. To verify this, molecular docking and molecular dynamics simulations were performed to predict the nature of the interaction and the dynamic behavior of the two compounds in the BSA complex, PS1 and PS2, under physiological conditions of pH = 7.1. In addition, the free energies of binding for the BSA-PS1 and BSA-PS2 complexes were estimated at 300 K based on the molecular mechanics of the Poisson–Boltzmann surface (MMPBSA) with the Gromacs package. PS2 was found to have a higher binding affinity than PS1. To determine the behavior of the orbital transitions in the ground state geometry, we found that both compounds have similar orbital transitions from HOMO–LUMO via π → π* and HOMO–1–LUMO+1 via n → π*, which was included in the FMO analysis. A cytotoxicity study was performed to determine the toxicity of the compounds. Based on the MD study, the stability of the compounds with BSA and the dynamic binding modes were further revealed, as well as the nature of the binding force components involved and the important residues involved in the binding process. From the binding energy analysis, it can be assumed that PS2 may be more active than PS1.

## 1 Introduction

Nowadays, multidrug resistance (MDR) is one of the challenges in treating bacterial infections. The continuous proliferation of MDR bacteria that evade antibacterial efficacy increases the likelihood of treatment failure.^1,2^ In this context, new drugs with novel agents with enhanced activity are urgently needed to combat the MDR problem. In this regard, dithiocarbazate compounds are more attractive options for investigation, and possible development as antibacterial and cytotoxic agents should be flexible functions. In recent decades, the chelating compounds of sulfur–nitrogen with the properties of cytotoxicity,^3,4^ antibiotics,^5^ antibacterial,^6^ anti-*Trypanosoma cruzi*,^7^ and anti-*Mycobacterium tuberculosis*^8^ from *S*-alkyl/aryl esters of dithiocarbazinic acid have been extensively explored. These and related Schiff bases^9−12^ continue to receive much attention due to the potential modification of their properties by the introduction of different substituents via the condensation of various S-substituted dithiocarbazate esters with numerous aldehydes and ketones. Based on this previous report,^13,14^ we synthesized the new compounds PS1 and PS2, which may have more useful functions against bacterial infections. Based on molecular docking studies, the binding free energy for *S*-benzyldithiocarbazate-based Schiff bases^15^ with proteins is higher than the case of our developed compound, the binding free energy for PS compounds; so, the binding affinity is stronger than that of the SBDTC Schiff base, the performance of our developed compound is finally active, and the binding affinity character with the chemical reactivity ratio is also improved based on the HOMO–LUMO energy gap analysis (Δ*E*_HOMO–LUMO_). Based on these facts, we extend our studies and use our analogy to protein-drug interactions, which gives a clear picture of the interaction with the human body. For this purpose, we first tried bovine serum albumin (BSA) because BSA serves as an ideal protein^16^ model due to its solubility in water and buffer medium and its remarkable binding properties with similar characteristics to human serum albumin (HSA). Moreover, many studies could be useful to understand the structural information on drug binding to albumin to determine the therapeutic effect of drugs. Consequently, BSA binding studies help to understand the importance of the drug-binding problem that arises in the field of life sciences and clinical medicine.

The study of protein–ligand interactions is of great benefit and has played an immense role in industrial, cosmetic, biological, and pharmaceutical applications.^17^ BSA is a large globular protein and represents a type of serum albumin that has been used as a reference protein for various studies. Its molecular weight is about 66000 Da,^18−21^ consists of 583 amino acid residues, and contains 17 disulfide bridges and one free SH group. It consists of three distinct homologous domains, I, II, and III, with each domain divided into two subdomains, A and B, respectively. It comprises two tryptophan residues, Trp-213 and Trp-134, with Trp-134 located in a hydrophilic environment near the protein surface (subdomain I B). Trp-213, on the other hand, is located in the largest hydrophobic well of domain II (subdomain II A). The major BSA binding sites are in subdomains IIA and IIIA.^22−25^

To determine the binding mode of drugs to proteins, various physicochemical techniques such as isothermal calorimetry and spectroscopy have been extensively used to study the binding of drugs to albumins.^26^ These experimental techniques are useful to determine the binding mode of drug interaction with proteins. In addition, in silico computational methods such as molecular docking and molecular dynamics simulations (MDS), which provide useful insights for drug discovery and development, shed light on the characteristic molecular mechanism between protein and ligand.^27^ Molecular docking is useful for determining the nature of the interaction of a protein–ligand complex. However, in the case of MDS, it is useful to predict the dynamic behavior of the protein when bound to substrate molecules at different time intervals.^28^ Molecular mechanics/Poisson–Boltzmann surface area (MMPBSA) is a molecular mechanics method in the continuum solvent approach to determine the free energies of the protein–ligand complex.^29^ Therefore, these simulation techniques are key to the methodological improvement of drug discovery and development. In these computational studies, which could complement the experimental study of spectroscopic methods by capturing the molecular interactions between bovine serum albumin (BSA) and pyrene derivatives (PYD), molecular docking, which is useful for predicting the binding mode between BSA and PS compounds, and the MDS method are useful for extracting the dynamic alignment of BSA-PS compounds in complex media in the solvent environment. In addition, the free energy of binding was estimated using the integrated methods MD and MMPBSA, which are useful for understanding the binding properties of PYD with BSA. To confirm the nature of the electronic transition of the compounds by analysis, we used HOMO–LUMO (highest-occupied molecular orbital–lowest-unoccupied molecular orbital), and the toxicity of the compounds was explained by computational cytotoxicity studies.

In this context, the present study using in silico simulations is well aligned with the experimental spectroscopic study of BSA with PYD to provide more insights into molecular interactions. In contrast, molecular docking studies are used with the binding mode of interaction between BSA and PYD, and even more is learned about the dynamic nature of BSA with PYD with MD simulations on different time scales. To improve our understanding of PYD with BSA, we also calculated the free energy with MD and MMPBSA. We also analyzed the toxic effects of these compounds (PS1 and PS2) using computational methods. This kind of study on antibacterial drugs could be useful for further investigation.

## 2 Experimental Section

### 2.1 Materials and Physical Measurements

All chemicals and solvents were reagent-grade and used without further purification. Reagents, such as pyrene-1-carbaldehyde and hydrazine hydrate, were obtained from commercial sources and used without further purification. The starting materials *S*-methyldithiocarbazate^30^ and *S*-benzyldithiocarbazate^31^ were prepared as previously reported. The final compounds *N*′-pyren-1-ylmethylene-hydrazinecarbodithioic acid methyl ester (PS1) and *N*′-pyren-1-ylmethylene-hydrazinecarbodithioic acid benzyl ester (PS2) were synthesized as previously reported.^32^ Elemental analyses were performed on a PerkinElmer Series II CHNS/O analyzer 2400. ^1^H and ^13^C NMR spectra were measured on a Bruker AVANCE II 400 MHz NMR spectrometer. Infrared spectra were recorded using a PerkinElmer 983 model FT-IR spectrophotometer with compounds dispersed as KBr discs. Electronic spectra were recorded on an Agilent-8453 diode array spectrophotometer. ESI-mass spectra were recorded using an Agilent 6200 series Q-TOF LC-MS instrument.

### 2.2 Synthesis of Ligands

**Scheme 1:**
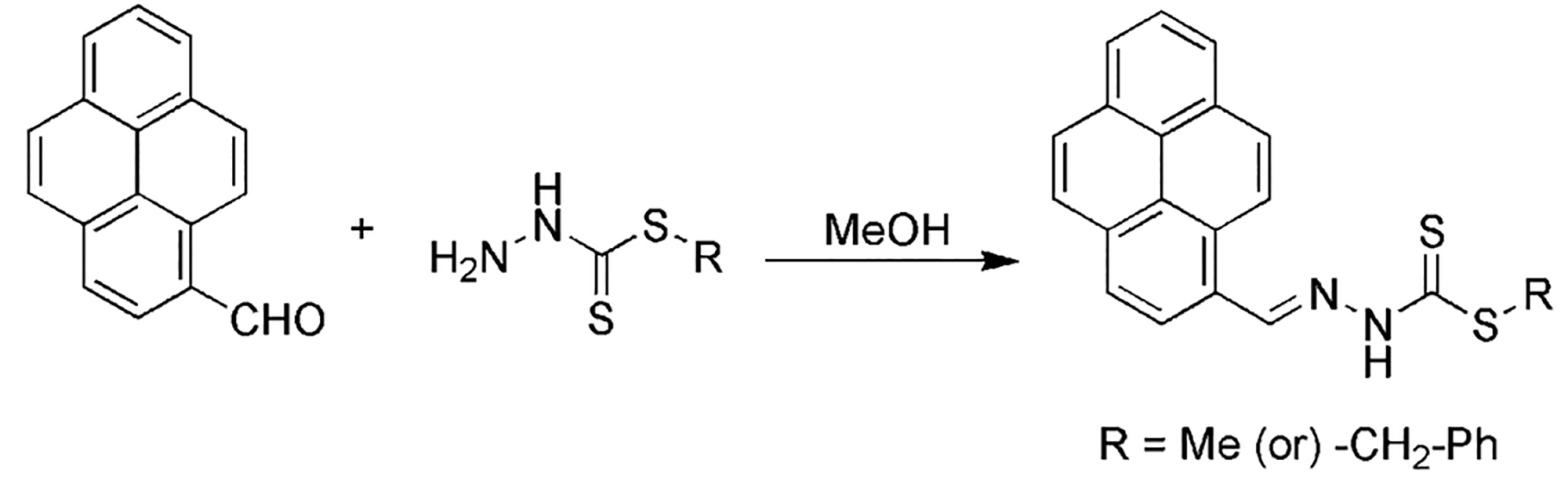
Synthesis of Pyrene Derivatives

*N*′-Pyren-1-ylmethylene-hydrazinecarbodithioic acid methyl ester (PS1): To a solution of *S*-methyldithiocarbazate (0.06 g, 0.5 mmol) in methanol (20 mL) was added a solution of 1-pyrenecarboxaldehyde (0.12 g, 0.5 mmol) in methanol (5 mL). When refluxing, a bright yellow precipitate was obtained. The mixture was allowed to stand at room temperature overnight, and the resulting yellow precipitate was filtered off and washed with cold methanol. It was recrystallized from methanol, and the yellow fluffy compound obtained was dried under a vacuum over P_2_O_5_ (Scheme 1). Yield: 68%. ^1^H NMR (DMSO-d_6_) δ (ppm) 9.22 (s, 1H, −CH=N−), 8.82–8.70 (d, *J* = 12 Hz, 1H, Ar–H), 8.46–8.44 (d, *J* = 8 Hz, 1H, Ar–H), 8.37–8.25 (m, 5H, Ar–H), 8.20–8.18 (d, *J* = 8 Hz, 1H, Ar–H), 8.13–8.09 (t, *J* = 8 Hz, 1H, Ar–H), 2.59 (s, 3H, S-CH_3_). ^13^C NMR (DMSO-d_6_) δ (ppm) 197.85 (C–S), 145.60 (C=N), 132.30, 130.64, 129.95, 129.10, 128.84, 128.73, 127.27, 126.61, 126.29, 125.96, 125.73, 125.63, 125.11, 124.00, 123.51. 122.37 (Ar–CH), 16.50 (S–CH_3_). ESI-MS (*m*/*z*): 335.0665 [M + H]^+^. Anal. Found (called) for C_19_H_14_N_2_S_2_: C, 68.29 (68.23); H, 4.19 (4.22); N, 8.43 (8.38).

*N*′-Pyren-1-ylmethylene-hydrazinecarbodithioic acid benzyl ester (PS2): The same procedure was followed for the preparation of compound L2 using *S*-benzyldithiocarbazate (0.09 g, 0.5 mmol) instead of *S*-methyldithiocarbazate. Yield: 70% ^1^H NMR (DMSO-d_6_) δ (ppm) 9.23 (s, 1H, −CH=N−), 8.70–8.68 (d, *J* = 8 Hz, 1H, Ar–H), 8.42–8.40 (d, *J* = 8 Hz, 1H, Ar–H), 8.34–8.22 (m, 5H, Ar–H), 8.16–8.14 (d, *J* = 8 Hz, 1H, Ar–H), 8.10–8.06 (t, *J* = 8 Hz, 1H, Ar–H), 7.47–7.45 (d, *J* = 8 Hz, 1H, Ar–H), 7.36–7.32 (t, *J* = 8 Hz, 2H, Ar–H), 7.29–7.25 (t, *J* = 8 Hz, 2H, Ar–H), 4.56 (s, 2H, S-CH_2_). ^13^C NMR (DMSO-d_6_) δ (ppm) 195.99 (C–S), 145.78 (HC=N), 136.86, 132.37, 130.68, 129.95, 129.22, 129.13, 128.97, 128.80, 128.50, 127.29, 127.25, 126.64, 126.33, 126.01, 125.54, 125.41, 125.16, 123.99, 123.49, 122.15 (Ar–CH), 16.50 (S–CH_3_). ESI-MS (*m*/*z*): 411.0979 [M + H]^+^. Anal. found (calcd) for C_25_H_18_N_2_S_2_: C, 73.22 (73.14); H, 4.49 (4.42); N, 6.75 (6.82).

### 2.3 In Silico Investigation

#### 2.3.1 Optimization of Pyrene Derivatives with DFT

To perform the molecular modeling studies, the molecular structures of PS1 and PS2 were first developed using Avogadro 1.1.1^33^ software. Geometry optimization for the developed molecular models was performed within the framework of density functional theory (DFT) with the B3LYP exchange-correlation functional with basis set 6-31 + G* using the Gaussian 09 package.^34^ The optimized geometry of PS1 and PS2 was confirmed by vibrational analysis to ensure that an energy-minimized conformation without imaginary frequency is possible.

#### 2.3.2 Molecular Docking of BSA with PYD

The crystal structure of BSA was extracted from the Protein Data Bank (PDB) (https://www.rcsb.org/) with a PDB-ID of 4f5s and a resolution of 2.47 Å. Chimera 1.8 software^35^ was used to remove the chain B, water, and other molecules from the PDB structure of BSA. To obtain the initial structure of the BSA-PS complex for molecular dynamics (MD), a joint protocol using the binding process for rigid protein structures and flexible drugs was performed with^36,37^ Auto Dock 4. Docking calculations were performed using Lamarck’s genetic algorithm (LGA)^38^ with PYD “torsion bonds” moving freely, while the BSA was in rigid mode. Autodocking tools were used to add hydrogen to the BSA protein, and partial charges were assigned to the Kollman unit atom.^39^ The size of the lattice map was set to (110 × 110 × 110) Å^3^ within the lattice spacing of 1.12 Å in the *x*, *y*, and *z* directions, where the program itself is defined. The stable conformation corresponding to the lowest binding energy was determined by docking analysis. Fifty independent runs were generated for a maximum energy evaluation of 25,00,000 and a population size of 150. Auto Grid 4 generated the energy grid maps for all ligand atom types before performing the docking calculation.

#### 2.3.4 Classical Molecular Dynamics (MD) Simulations

All MD simulations and trajectory analyzes were performed with the Gromacs 2020.2 software package^40^ using the AMBER99SB force field with the TIP3P water model.^41^ The BSA-PS complex was first energetically minimized using the steepest descent method with 3000 steps and then with 2000 steps to minimize the energy of the conjugate gradient. In addition, the system is well balanced with 1000 ps MD for the position constraint of the BSA system with PYD to allow the relaxation of the water molecules. The equilibrium runs after 1 ns for the system without position constraint to allow the system to reach an equilibrium level. The root mean square deviation (RMSD) was useful for monitoring the equilibrium system around the initial reference structure, the electrostatic term was explained by the Ewald algorithm for the particle network, and the LINCS^42^ algorithm for confining all heavy atoms except hydrogen, the SETTLE^43^ algorithm for the water molecule, the dielectric permittivity of ε = 1 at the 2 fs time step, and the Maxwellian distribution for the initial velocity at the initial temperature of 300 K during the first equilibrium run were used to fit the density of the system. During the NPT step, the weak coupling constant (*P*_0_ = 1 bar) was used (the coupling constant = *T*_p_ = 0.5 ps). A cutoff point of 0.25 nm^44^ was used for cluster structure analysis. Antechamber^45^ was used to apply the parameters to the two compounds. The AM1-BCC method was used to calculate the partial atomic charges of the PYD. The RESP^46^ charges were applied to all atoms calculated at the HF/6-31+G*^47^ theory levels using Gaussian 09^34^ software. Trajectory analysis was performed using the VMD and Gromacs analysis tools.

#### 2.3.5 Calculation of Bond-Free Energy

In this study, we performed binding free energy calculations using molecular mechanics/Poisson–Boltzmann surface^48^ with Gromacs’ g_mmpbsa^49^ tools. We selected a total of 200 snapshots from MD trajectories, where each snapshot was selected from all 200 ps in a total of 4000 configurations.

The calculation of binding free energies is based on formula 1:
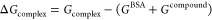
1Here *G*_complex_, *G*^BSA^, and *G*^compound^ are the free energies of binding of the complex, BSA, and PS compounds, respectively. Each term can be expressed by formulas 3 and 7
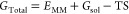
2where *E*_MM_ is the molecular mechanics term, *G*_sol_ is the solubility free energy, and TS is the conformational entropy upon binding of the ligand. This has been ignored due to the high computational cost and low predictability.^50^

#### 2.3.6 Molecular Mechanics Potential Energy

Analysis of the decomposition of the free energy of the potential energy term of molecular mechanics: The potential energy for the vacuum, *E*_MM_, is composed of the energy of the bond and nonbonded interactions, and the energy values were determined from the parameters of the molecular mechanic’s force field (MM).^51^
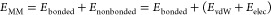
3Here, *E*_bonded_ includes the energy terms of the bond, angle, dihedral, and improper interactions. *E*_nonbonded_ gives both the electrostatic (*E*_elec_) and van der Waals (*E*_vdW_) interactions by calculating the terms of the Coulomb and Lennard-Jones potential functions, respectively. The single trajectory approach assumes that the bound and unbound conformations of protein and ligand are identical. Therefore, *E*_bonded_ is always equal to zero.^52^

The electrostatic term *E*_elec_ is meant for the electrostatic interactions of different (oppositely charged) atomic particles, and each atom considered a single point particle is calculated using eq 4
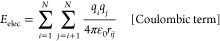
4where *q*_*i*_ and *q*_*j*_ are the atomic partial charges (e), *r*_*ij*_ is the interatomic distance (Å), and *E*_vdW_ is the van der Waals term.

The atoms interact without bonding with a relatively weak attractive force acting on the neural atoms or molecules due to the presence of other particles, inducing the electrical polarization of the individual particles. The energy of the term is calculated using eq 5 in a molecular mechanics force field
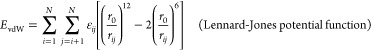
5where ε_*ij*_ is represented as the interaction strength (kcal/mol), and *r*_0_ is the van der Waals radius (Å).

The molecular mechanism is described by eq 6:
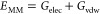
6Here, the solvation energy is estimated from the polar and nonpolar terms of the contribution conditions, as shown in eq 7
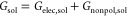
7

## 3 Results and Discussion

### 3.1 Synthesis and Characterization of Pyrene Derivatives

The compounds *N*′-pyren-1-ylmethylene-hydrazinecarbodithioic acid methyl ester (PS1) and *N*′-pyren-1-ylmethylene-hydrazinecarbodithioic acid benzyl ester (PS2) were prepared by condensation reactions of 1-pyrenecarboxaldehyde under reflux conditions of the corresponding S-substituted dithiocarbazates. The bright yellow solid obtained was recrystallized from methanol, giving soft yellow solids that are air-stable and highly soluble in polar aprotic solvents. The compounds were further characterized by several spectroscopic methods, and their purity was confirmed by elemental analysis.

**Scheme 2:**
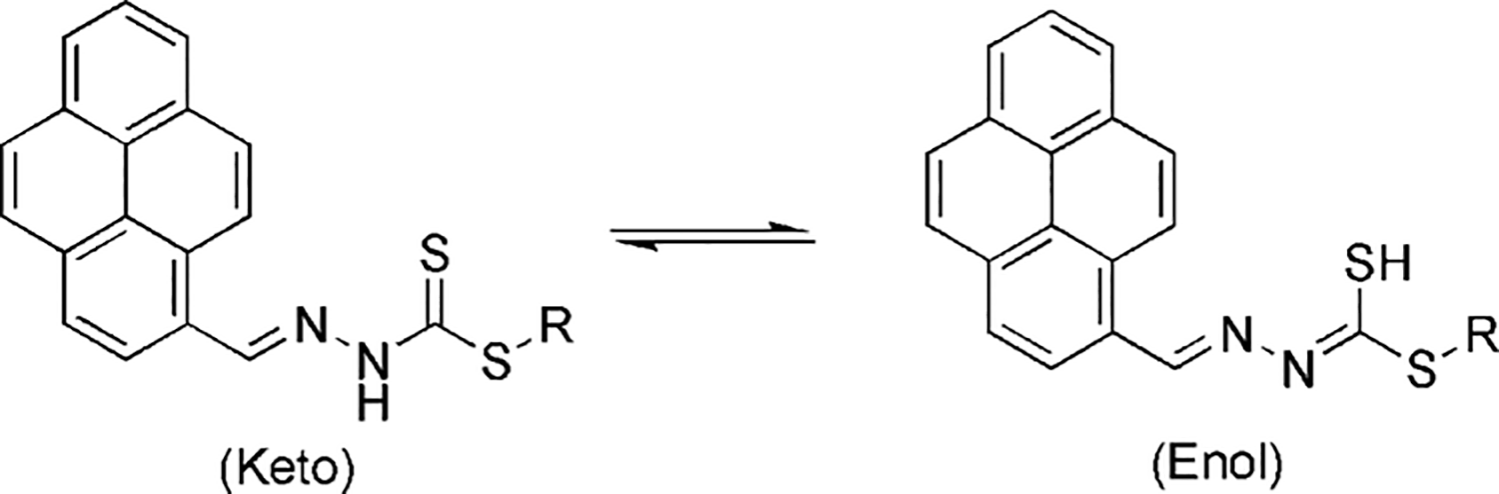
Thione (Left) and Thiol (Right) Forms of the Compounds

The compounds can exist as thione or thiol tautomers or as a mixture of both tautomers (Scheme 2). In the solid state, FTIR indicated that the compounds are primarily in the form of a thione tautomer due to the presence of a υ(NH) band at 3089 cm^–1^ and the absence of a υ(S–H) band around 2600 cm^–1^. The ESI mass spectra show molecular ion peaks at *m*/*z* [M + H]^+^ (PS1: 335.0665, PS2: 411.0979) that correspond to the proposed structures of all compounds.

**Figure 1:**
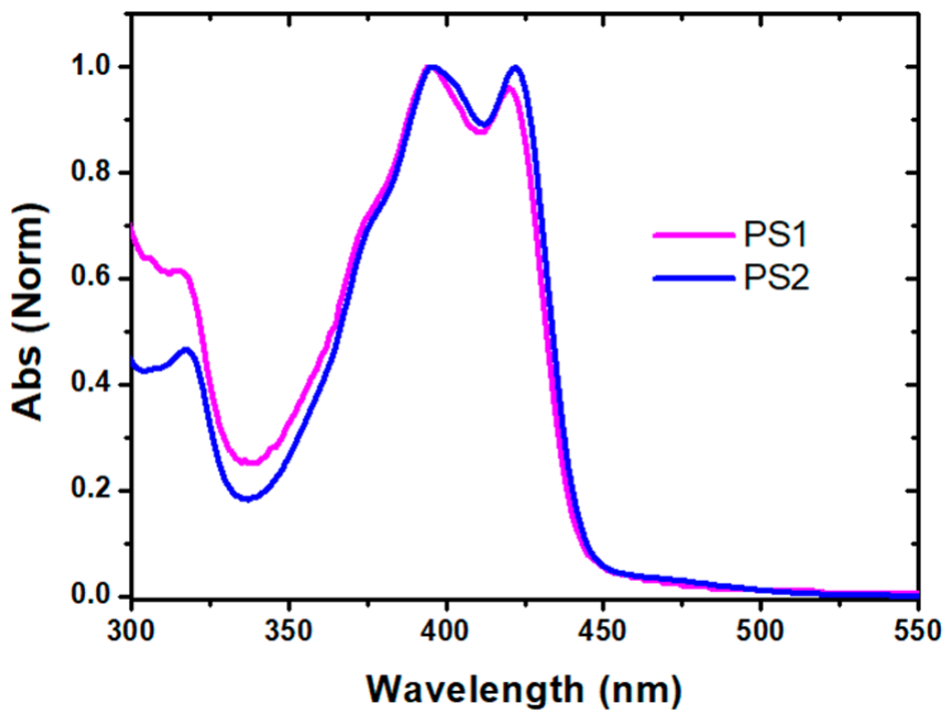
Normalized absorption spectrum of pyrene derivatives in DMSO.

The absorption spectrum of pyrene derivatives in the UV–vis range was measured and shown in Figure 1. The figure showed distinct pyrene absorption bands at 315, 395, and 420 nm. The absorption at 315 nm was attributed to π–π* transition, while the longer wavelength bands (395 and 420 nm) were attributed to n−π* transitions of imine functionalized pyrene units. Intriguingly, the spectral behavior of both derivatives was extremely similar.

### 3.2 Computational Analysis

#### 3.2.1 Optimized Geometry and FMO Analysis

**Figure 2:**
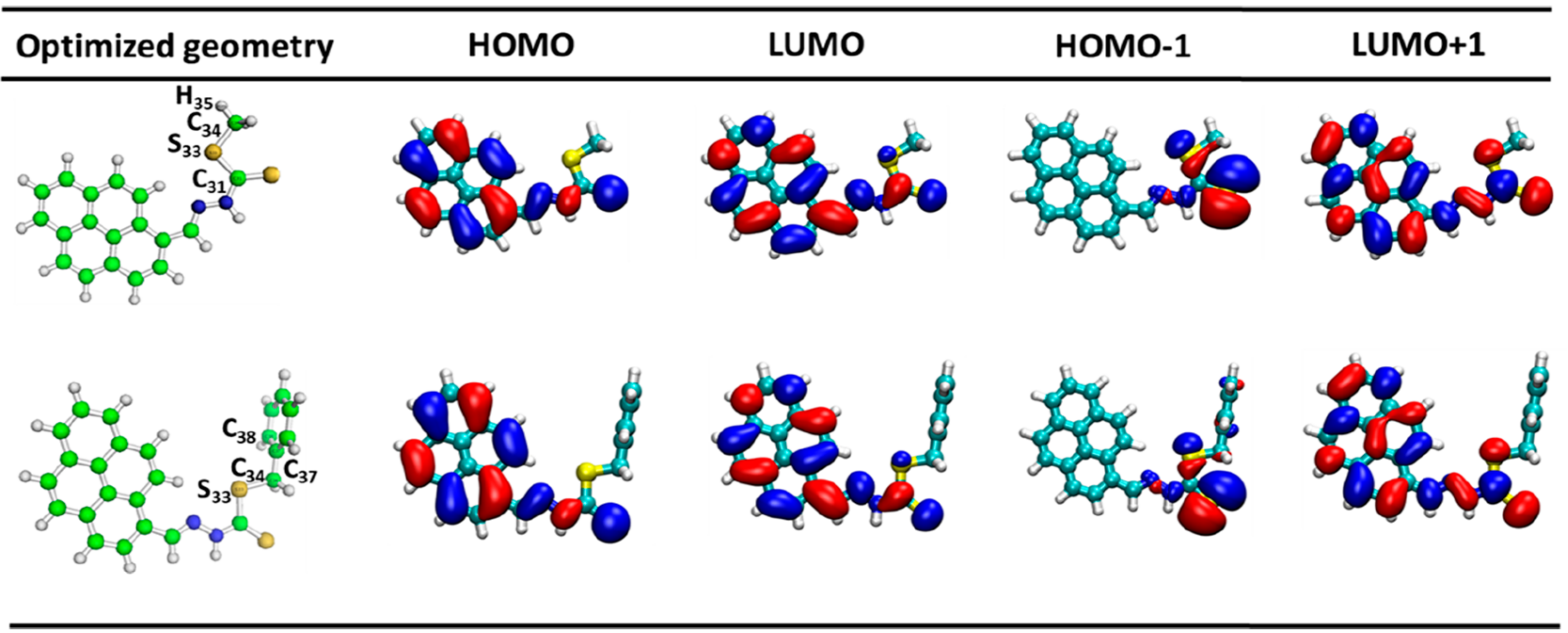
Optimized geometry and HOMO/LUMO levels of PS1 (top) and PS2 (bottom) compounds.

The geometry-optimized compound PS1 is a planar geometry and PS2 is a nonplanar geometry since the dihedral angle of the atoms of C_31_–S_33_–C_34_–H_35_ was measured to be 180° for PS1 and 90° for S_33_–C_34_–C_37_–C_38_ in PS2. The terminal methyl group of the hydrogen atom of PS1 is replaced by a phenyl ring perpendicular to the pyrene ring, which is designated as PS2. Various organic molecules of the previously reported electronic transitions, such as π → π*, n → π*, and π(donor) → π*(acceptor),^53^ can be seen in Figure 2, which shows the molecular FMO representation of both PS1 and PS2 compounds. With this representation, we could observe the electronic FMO transitions for both compounds obeying the HOMO → LUMO and HOMO–1 → LUMO+1 transitions as π → π* and n → π*, respectively. The charge moieties of PS1 are only on the π-ring of HOMO; to spread over the ring of LUMO orbitals of π → π* and for HOMO–1 to LUMO+1 transitions, only nonbonding orbitals (n) are on the π-ring side of the molecule, as for the n → π* transitions. A similar behavior observed for PS2 is shown in Figure 2. Although two electronic states are involved in both compounds, the π–π* has the bright state in both cases, the intensity of PS1 and PS2 compounds is 0.8771 and 0.8477 for π–π*, but the n−π* has the zero-intensity state in both compounds. The energy gap (HOMO–LUMO) of the PS1 compound is 0.1140, and PS2 has an energy gap of 0.1139, almost identical values in both cases as shown in Figures S1 and S2 (Supporting Information). In this scenario, based on the report,^15^ the chemical reactivity of the compounds is also improved, which is directly related to the HOMO–LUMO energy gap (Δ*E*_HOMO–LUMO_), which is smaller than those of *S*-benzyldithiocarbazate-based Schiff bases (2.43 eV), highlighting that the properties of our developed compounds are improved for multiple applications. In this context, it could be suggested that both PS1 and PS2 have similar reaction-promoting media in chemical reactions.

#### 3.2.2 Fluorescence Quenching Measurements

**Figure 3:**
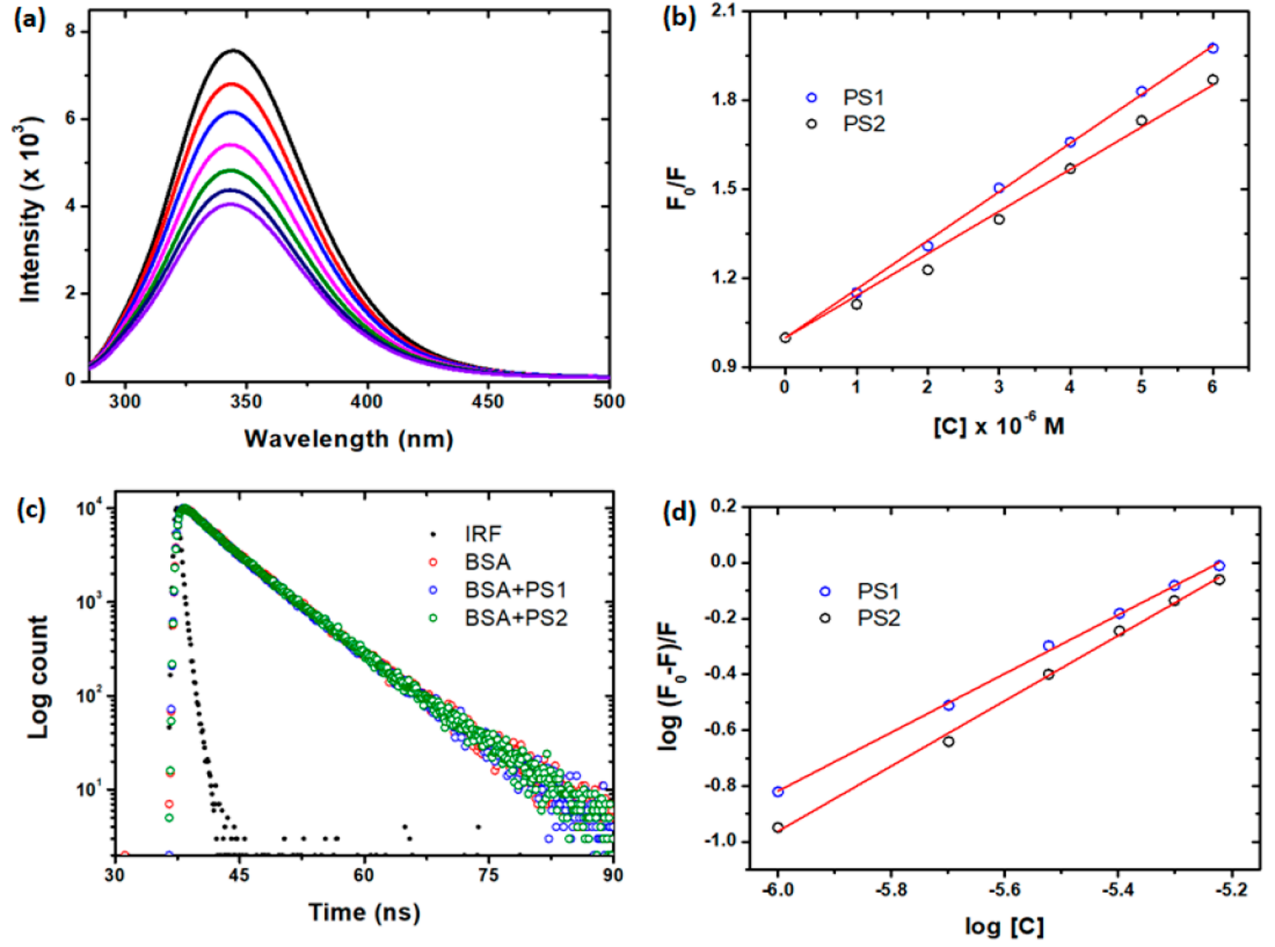
(a) BSA fluorescence quenching spectra with various concentrations of PS2, (b) Stern–Volmer plot for fluorescence quenching, (c) time-resolved fluorescence decay of BSA and its complexes (λ_exi_: 280 nm and λ_emi_: 340 nm), and (d) plot between log(*F*_0_–*F*)/*F* and log [*Q*].

The fluorescence quenching method was commonly used to evaluate the molecular interaction of small or drug molecules with BSA. In this context, we have adapted the same method to investigate the interaction of new pyrene derivatives with the BSA protein. Figure 3a depicts the influence of PS2 on the fluorescence spectra of BSA. The fluorescence intensity of BSA steadily decreases as the concentration of pyrene derivatives increases, according to our findings. A similar type of fluorescence quenching was observed with PS1, and spectra were not shown here. Fluorescence intensities were corrected for the inner filter effect, according to the following equation
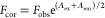
8where *F*_cor_ is the corrected fluorescence intensity, *F*_obs_ is the observed fluorescence intensity in the presence of complexes, and *A*_ex_ and *A*_em_ are the total absorbances at the excitation wavelength (λ) and the emission wavelength (λ), respectively. Dynamic quenching, static quenching, and a combination of both are the most common types of fluorescence quenching. The fluorescence quenching data were examined with the Stern–Volmer equation (eq 9) to predict the possible quenching mechanism of pyrene derivatives with BSA
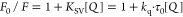
9where *F*_0_ and *F* are the fluorescence intensities of BSA in the absence and presence of pyrene derivatives, [*Q*] is the concentration of pyrene derivatives, *K*_SV_ is the Stern–Volmer constant, *k*_q_ is the quenching rate constant, and τ_0_ is the fluorescence lifetime of BSA (6.05 ns). A typical Stern–Volmer plot of BSA quenching by pyrene derivatives is shown in Figure 3b. Interestingly, a linear plot for pyrene complexes is obtained by plotting *F*_0_/*F* and [*Q*]. From the slope of the linear graph (Figure 3b), the *K*_SV_ and *k*_q_ values were determined and listed in Table 1. The obtained *k*_q_ values are substantially more significant than the maximal collisional quenching rate constant (10^10^), indicating that BSA quenching by pyrene derivatives is primarily due to the static quenching pathway. In addition, we performed time-resolved measurements of fluorescence decay to confirm static quenching. If the quenching is caused by dynamics or FRET, the fluorescence lifetime of BSA should be reduced. On the other hand, if the quenching is caused by static, the lifetime of BSA remains unchanged. Figure 3C shows the fluorescence decay of BSA in the absence and presence of pyrene derivatives. The fluorescence decay of BSA remains unchanged after interaction with pyrene derivatives, and this figure shows that the quenching is due to a static process.

**Table 1:** Stern–Volmer Quenching Constants (*K*_SV_), Quenching Rate Constants (*k*_q_), Binding Constants (*K*_b_), and Binding Sites (*n*) for the Interaction between BSA and Pyrene Derivatives

molecules	*K*_SV_ × 10^5^ (M^–1^)	*k*_q_ × 10^13^ (M^–1^ s^–1^)	*K*_b_ × 10^5^ (M^–1^)	*n*
PS1	1.64	2.71	7.39	1.05
PS2	1.42	2.34	7.81	1.16

Since the quenching of BSA inferred by binding of pyrene derivatives is a static process, the apparent binding constant, *K*_a_, and the number of binding sites, *n*, were calculated using eq 10.
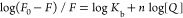
10

The linear graphs of log(*F*_0_–*F*)/F vs *n* log[*Q*] are shown in Figure 3d. The intercept and slopes of the double logarithm curves, as shown in Figure 3d, were used to derive the values of *K*_b_ and *n*. The *n* values were calculated, all-around one, indicating only one binding site for the pyrene derivatives.

The spontaneity of the interaction between BSA and pyrene derivatives can be analyzed with the standard Gibbs free energy (eq 11)
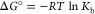
11where *R*, *T*, and *K*_b_ refer to the gas constant (8.314 J K^–1^ mol^–1^), absolute temperature (298 K), and binding constant, respectively. The calculated Δ*G*° values for PS1 and PS2 are −33.47 and −33.59 kJ mol^–1^, respectively. The negative Δ*G*° values indicate that the binding processes for both derivatives are spontaneous. Accordingly, the entropy change of the system is absolutely positive (Δ*S* > 0). Therefore, the quenching of BSA fluorescence induced by pyrene derivatives is driven by hydrophobic interactions.^54^

#### 3.2.3 In Silico Docking Analysis

**Figure 4:**
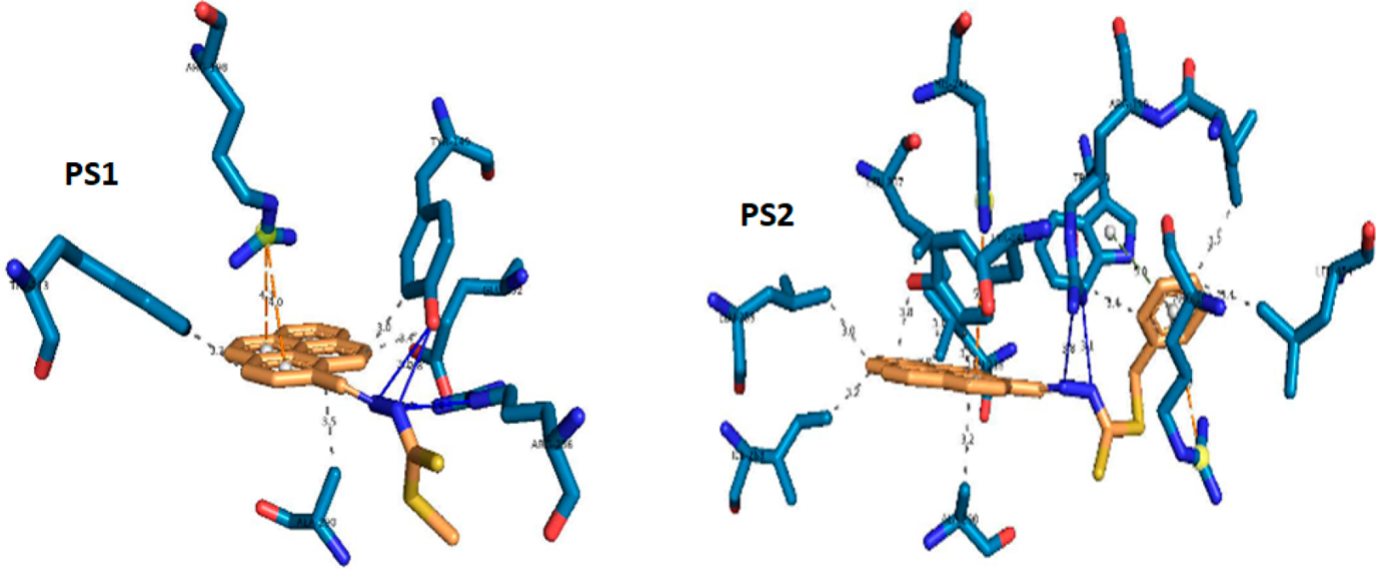
Docking positions for both compounds of PS1 and PS2.

The binding affinity of compounds PS1 and PS2 to the BSA protein was determined by docking studies. The calculated binding energies of PS1 and PS2 are −7.44 and −8.64 kcal/mol, respectively (see Table 2). The binding energy of PS2 has lower energy than that of PS1. The difference in binding energy could be related to the presence of the dominant force of the interactive term (vdW+Hbond+desolv energy) in PS2 compared with PS1 (−10.37 and −8.50 kcal/mol), although PS1 has two hydrogen bonds, leading to stronger binding affinity in the binding pocket; but PS2 has a more hydrophobic part (benzene ring) than PS1, which means that the hydrophobic interaction possibilities are more dominant in PS2 than in PS1, leading to higher binding affinity with lower values of binding free energy. The hydrogen bonding residues were Arg256 and Tyr149 for PS1 and Arg198 for PS2. Figure 4 shows the preferred binding position of BSA with PS1 and PS2.

**Table 2:** Docking Data for the Two Compounds PS1 and PS2

S. no.	protein	compd	RMSD (Å)	binding energy (kcal/mol)	inhibition constant (*K*_i_)	no. of H-bonds (BSA-PS complex)	amino acid involved in an interaction
1.	BSA	PS1	107.031	–7.44	3.50 μM	2	Arg256(A), Tyr149(A)
2.		PS2	108.18	–8.64	461.34 nM	1	Arg198(A)

**Figure 5:**
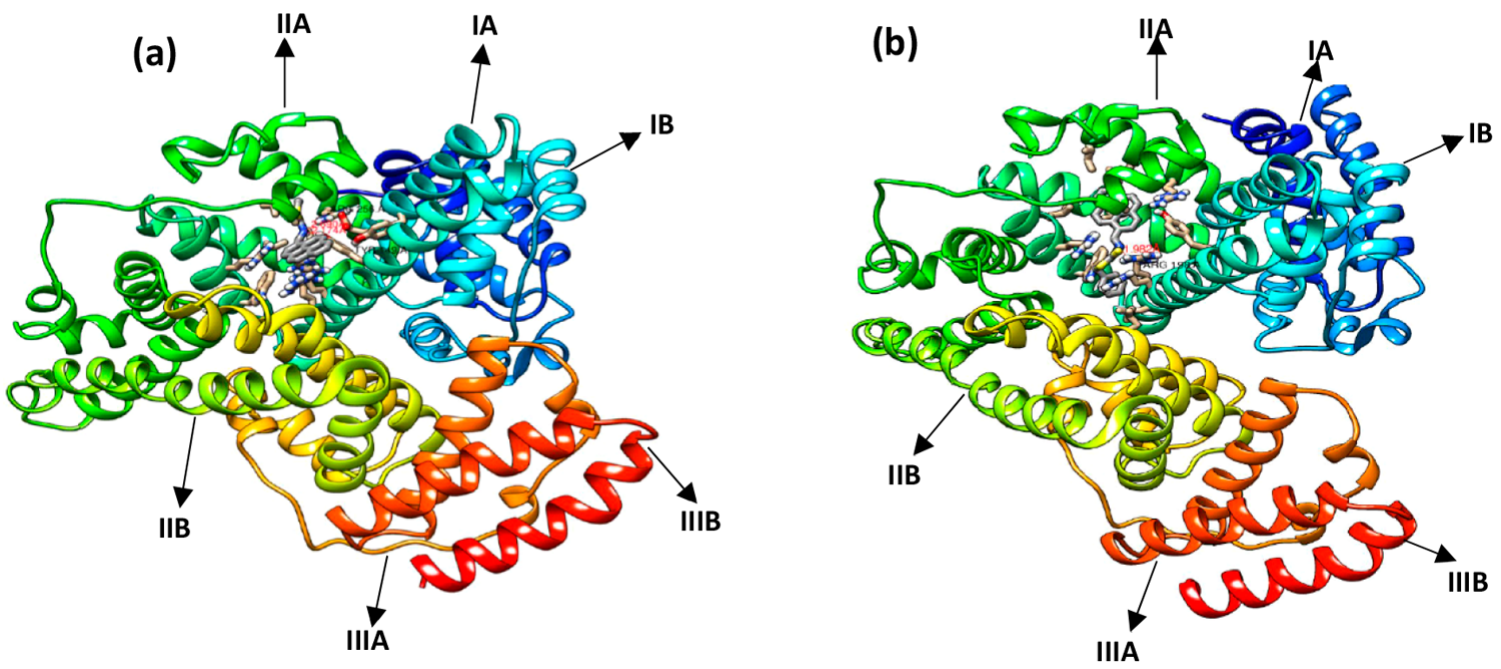
Interaction of BSA with (a) PS1 and (b) PS2.

The binding pockets of the BSA-PS complex are Arg198, Trp213, Arg217, Leu218, Phe222, Leu233, Leu237, Val240, Arg256, Leu259, Ile263, Ser286, Ile289, and Ala290. The hydrogen bonds between BSA and PS1 and PS2 consist of residues Arg256, Tyr149, and Arg198. Apart from the hydrogen bonds, the other interactions (hydrophobic interactions) are listed in Tables 3 and 4. BSA-PS1 shows the hydrogen bonds at “N” of Arg256 and “H” of PS1, “O” of Tyr149 and “H” of PS1 (Figure 5a), and “N” of Arg148 and “H” of PS2 (Figure 5b). The molecular docking results showed that in addition to hydrogen bonding and hydrophobic interactions, there were also π-cation interactions in PS1 and π–π interactions in PS2. The inhibition constants for both complexes are calculated according to formula 12
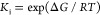
12Here, *R* is the universal gas constant (1.985 × 10^–3^ kcal mol^–1^ K^–1^), and *T* is the temperature (298.15 K) (see ref (55)).

**Table 3:** Hydrophobic Force between BSA of PS1 and PS2

complex	residue	amino acid	distance (Å)
BSA_PS1	149A	TYR	3.00
BSA_PS2	152A	GLU	3.37
	213A	TRP	3.31
	290A	ALA	3.54
	149A	TYR	3.68
	194A	ARG	3.50
	197A	LEU	3.16
	213A	TRP	3.38
	218A	LEU	3.18
	237A	LEU	3.13
	237A	LEU	3.76
	259A	LEU	3.02
	263A	ILE	3.24
	290A	ALA	3.22
	454A	LEU	3.35

**Table 4:** Hydrogen Bonds between PS1 and PS2 Compounds

complex	residue	amino acid	distance H–A (Å)	distance D–A (Å)
BSA-PS1	149A	TYR	2.26	2.27
BSA-PS2	149A	TYR	2.17	3.08
	256A	ARG	2.37	3.37
	256A	ARG	2.67	3.60
	198A	ARG	3.12	3.75
	198A	ARG	2.16	3.11

The values of inhibition constants for the complex as BSA-PS1 and BSA-PS2 are 3.50 μM and 461.34 nM, respectively (shown in Table 2).

#### 3.2.4 Studies in Molecular Dynamics

**Figure 6:**
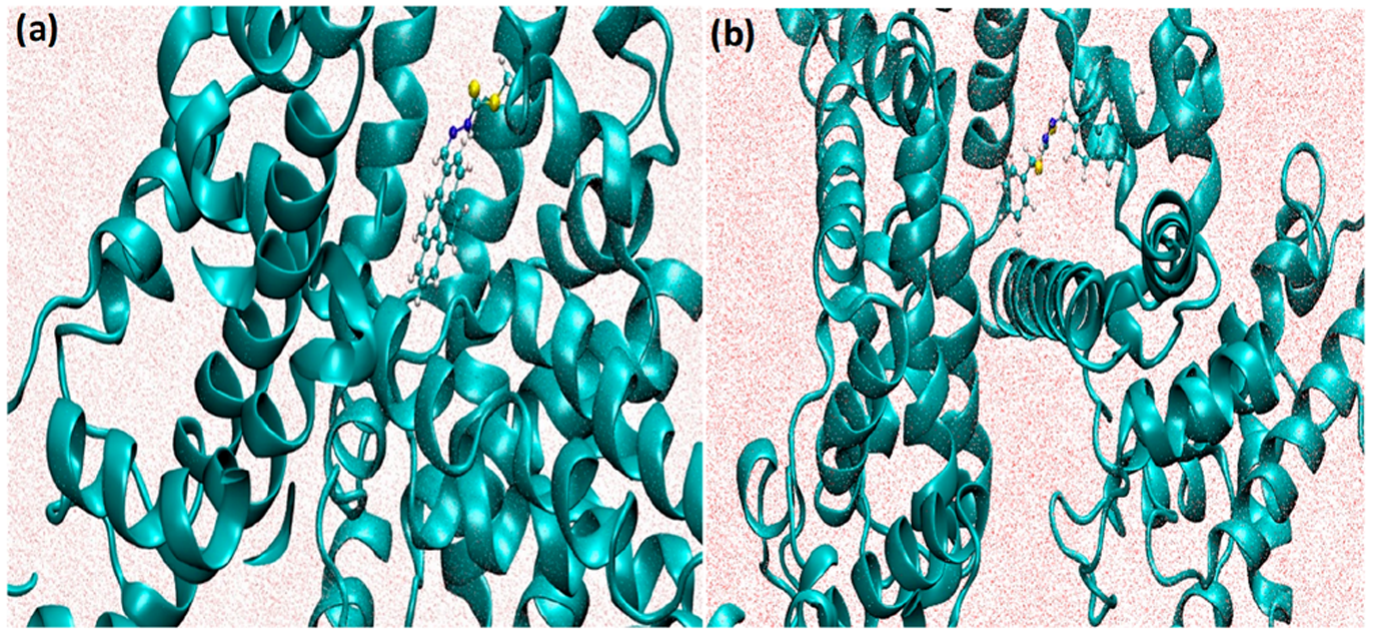
Conformational alignment of (a) PS1 (b) PS2 in complex with BSA.

Molecular dynamics (MD) is a well-known computer simulation method for the analysis of protein–ligand interactions that integrates the stability and energetics of the system at each time point. In the present study, MD was used to study the interaction between the BSA protein and two different compounds, such as PS1 and PS2 (Figure 6). We performed a simulation of up to 40 ns for the two compounds at 300 K and analyzed the trajectories for the stability of the system and the binding site.

#### 3.2.5 Analysis of Dynamic Binding Modes

Since MD simulations are the known tools to determine the flexibility and conformational alignment of BSA upon binding with compounds (drugs), the snapshots of the conformation of the BSA-PS complex were extracted from the simulations at time intervals of 15, 20, 30, and 40 ns for both compounds and analyzed to reveal the dynamic nature of the interactions between PYD and BSA. The total of four snapshots of each compound clearly shows that the PS1 and PS2 compounds are oriented differently in the IIA subdomain of BSA covered by different residues, as shown in Figure S3 and Figure S4. The conformational changes of the PS compounds are seen in the overlapping map of the four snapshots in Figure S5(a) and Figure S6(a). The average mean square fluctuation of the PS compounds shows that each atom fluctuates differently concerning different time scales at 15, 20, 30, and 40 ns for PS1 in Figure S5(b) and PS2 in Figure S6(b). The conformational changes of the PS compounds with BSA at four snapshots were further calculated and listed in Table S1. Analysis of the snapshots shows that the RMSD values vary from 15 to 40 ns when the no fit RMSD option is used. This analysis of the binding modes of the BSA-PS1 and BSA-PS2 complexes agrees well with the docking studies.

#### 3.2.6 RMSD and RMSF Analysis

**Figure 7:**
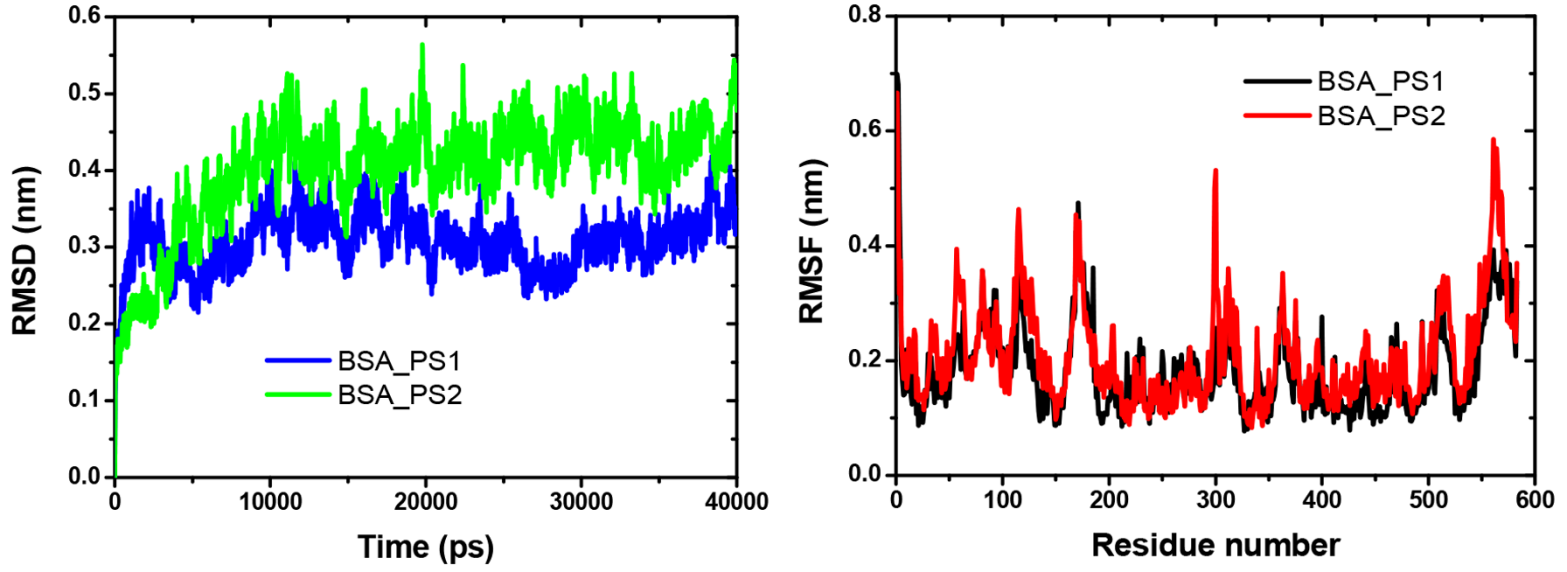
a: RMSD diagram for both compounds. b: RMSF fluctuations for both complexes.

The coordinates of the two complexes (BSA_PS1 and BSA_PS2) in the simulation are compared with the original reference structure of coordinates. From the root mean square deviation (RMSD) between the Cα atoms of the backbone of the coordinated complexes of PS1 and PS2 at 300 K, it is found that the system reaches equilibrium for both complexes starting from 1 ns, respectively. The BSA-PS2 complex is comparatively more stable than the PS1 complex due to the stronger interaction of BSA in PS2 than in PS1, the sterile factor of more hydrocarbons (benzene) also presents in the PS2 compound, and the RMSD deviation is at 3.0 Å for PS1 and at 4.0 Å for PS2; the deviations are shown in Figure 7a. The RMSF analysis of the complex and fluctuation residues is similarly effective, up to a 40 ns trajectory profile for the residues of the BSA-PS1 and BSA-PS2 complexes, as shown in Figure 7b.

#### 3.2.7 The Gyration Radius and the Interaction Energy

**Figure 8:**
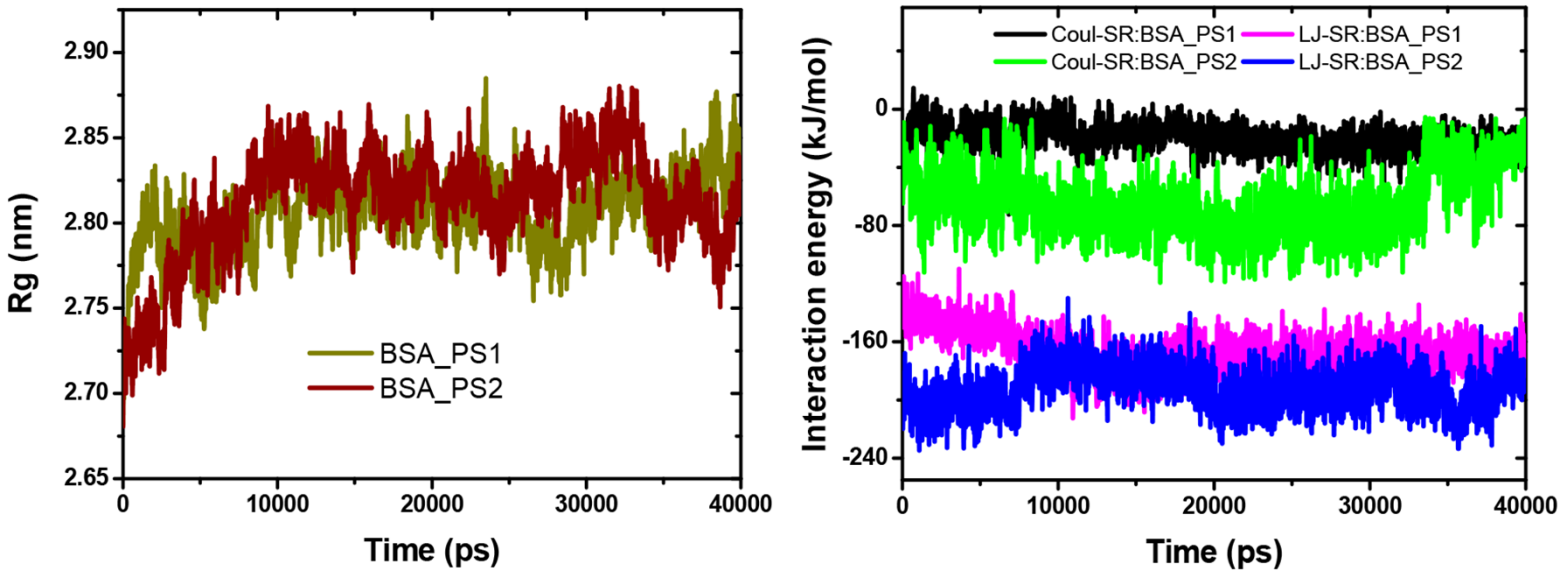
a: The gyration radius for both complexes. b: Interaction energy for both compounds.

The plot of the gyration radius shows that BSA, when bound in both PS1 and PS2, is always folded until the total simulation is up to 40 ns. This indicates that the compounds bind strongly to the BSA protein, as shown in Figure 8a. The interaction energy indicates how strongly the BSA protein interacts with the PYD. In this case, the BSA-PS2 complex has lower interaction energy than the BSA-PS1 complex, indicating that BSA-PS2 is more strongly bound than BSA-PS1 (see Figure 8b). The average interaction energies of Coulomb energy and Lennard-Jones energy of BSA-PS1 and BSA-PS2 are (−19.88 and −167.0 kcal/mol) and (−63.01 and −192.42 kcal/mol), respectively.

#### 3.2.8 Hydrogen Bonds and Bond Energy

**Figure 9:**
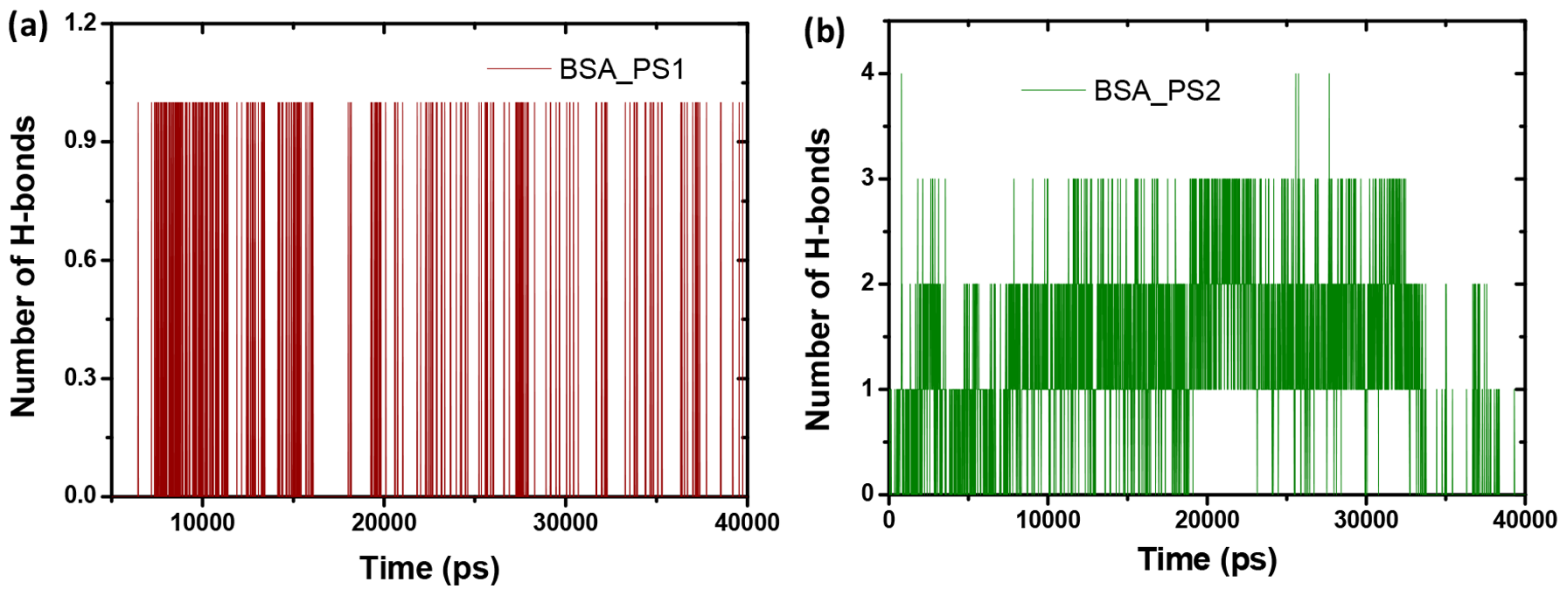
Hydrogen bonds for (a) BSA-PS1 and (b) BSA-PS2.

The three hydrogen bonds of the BSA-PS2 complex are present, but in the case of BSA-PS1, only one has up to 40 ns, which is due to the hydrophobic part of the hydrogen interacting with the protein rather than the less hydrophobic region shown in Figure 9a. The binding energy was estimated using the g_mmpbsa tool. The results were extracted and compared with the experimental free energy of binding given by the inhibition constant *K*_i_. Therefore, the reversible competitive inhibitors of the inhibition constant corresponding to the dissociation constant (*K*_d_)^56^ were estimated using the following formula.^57^
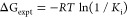
13Here, *R* and *T* are the constants of the gas and temperature, respectively.

**Figure 10:**
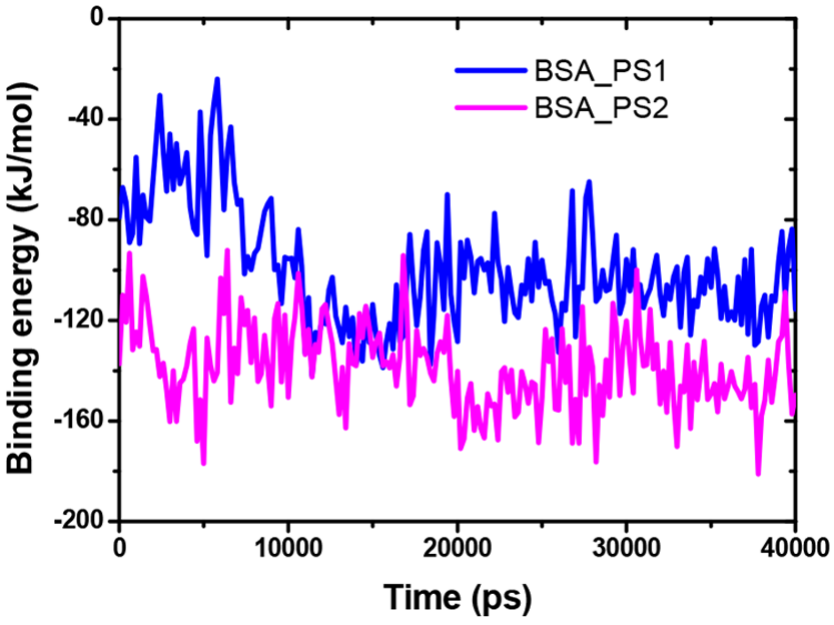
Binding free energies for both complexes.

**Figure 11:**
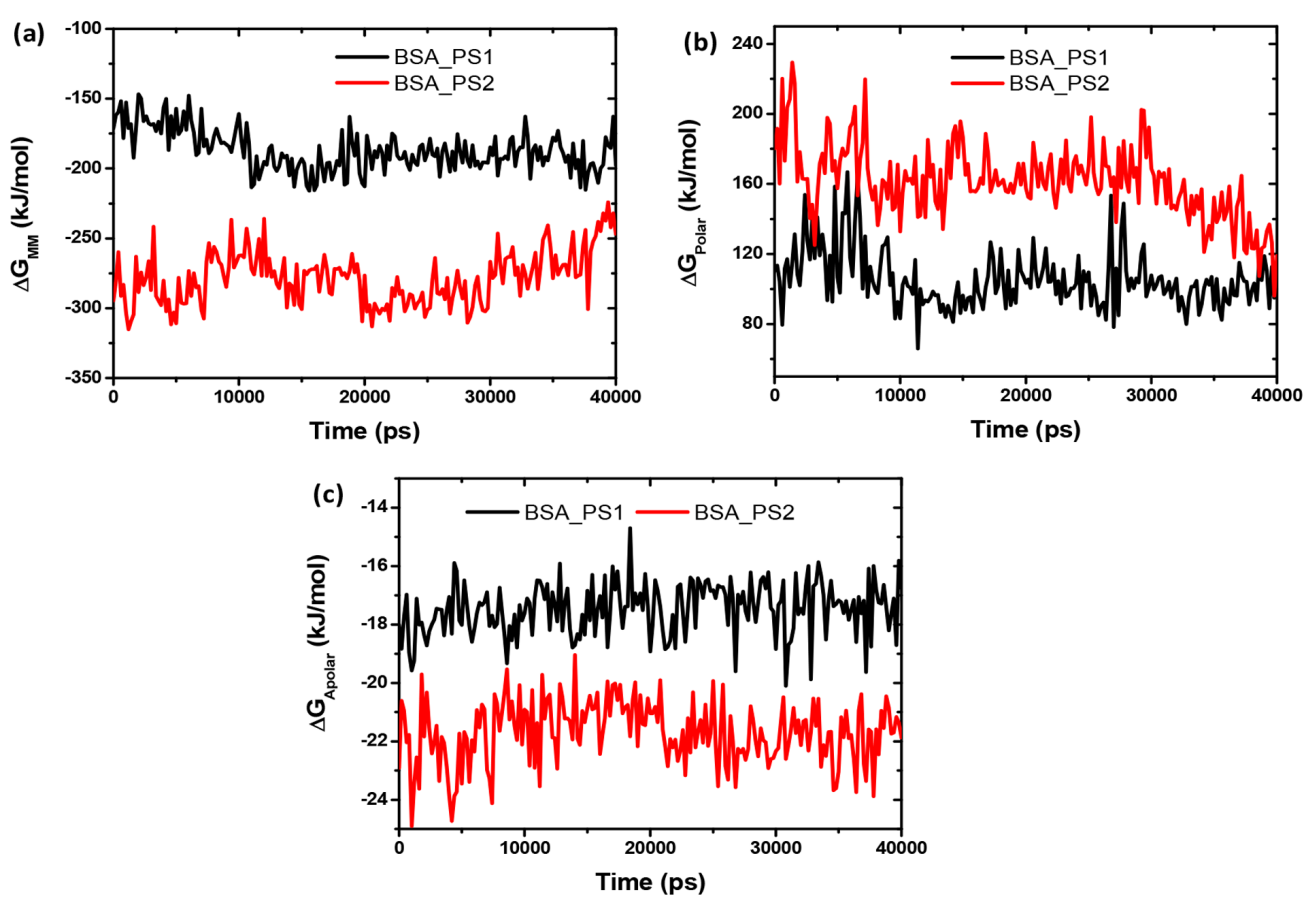
Energy components (a) Δ*G*_MM_, (b) Δ*G*_polar_, and (c) Δ*G*_apolar_.

In this case, BSA-PS2 has the lowest binding free energy −138.50 kJ/mol, but BSA-PS1 has only −99.30 kJ/mol, up to 40 ns extracted from 200 snapshots of MD trajectories (in Table 5). This means that the sequence of binding by BSA-PS2 > BSA-PS1 should be described in Figure 10. Thus, the results presented indicate that docking and MD yield the same pathway. The energy components of *E*_MM_, *G*_apolar_, and *G*_polar_ with each complex were calculated over time for 200 snapshots of 40 ns from the MD production run, and *E*_MM_ was estimated from LJ and the Coulomb potential. *G*_polar_ was calculated using a box created with the extreme coordinates of the complex in each dimension. Then, the box was expanded twice in each dimension to obtain the coarse-mesh box. During these extreme conditions in each direction, a 0.150 M NaCl salt solution with radii of 0.95 and 1.81 for sodium and chloride ions, respectively, was used for all *G*_polar_ calculations. The dielectric constants for vacuum and solvent were assumed to be 1 and 80, respectively. The value of *G*_apolar_ was estimated based on different nonpolar models using the parameters mentioned in ref (58), followed by the binding energy for each snapshot using a combination of eqs 1 and 2. In this case, the entropy calculation was not included in the binding free energy. All energy components are shown in Figure 11, and the values are listed in Table 6.

**Table 5:** Binding Free Energies for the Two Complexes

complex	binding free energy (Δ*G*) in kJ/mol
BSA_PS1	–99.30(1.6435)^a^
BSA_PS2	–138.50(1.1804)^a^

a States that standard mean error.

**Table 6:** Energy Components Determined for PS1 and PS2

energy components (kJ/mol)	PS1	PS2
Δ*G*_MM_	–180.10	–260.25
Δ*G*_polar_	–100.01	–250.0
Δ*G*_apolar_	–18.01	–23.0

#### 3.2.9 Cytotoxicity

Both PYD molecules were geometrically optimized with the Gaussian 09 package with density functional theory (DFT) using the functional B3LYP method of the base set 6-31+G*;^34^ ionization energy and the electron affinity were calculated according to the following formulas (eqs 14 and 15).
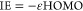
14
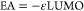
15

Ionization energy and electron affinity were used to calculate global properties such as electronegativity (χ), softness (S), hardness (η), electrophilicity index (ω), and chemical potential (μ). The values obtained from the calculated properties are shown in Table 7 where 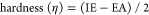
16
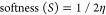
17
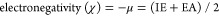
18
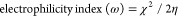
19From these observed values (in Table 7), we can conclude that a higher softness value indicates that the compounds have greater reactivity at this point, which means that the reactivity is greater, which subsequently leads to greater potency and a cytotoxic effect^59,60^ of the compounds. In this case, PS2 has a higher softness value of 8.7873 than PS1’s softness value of 4.3871; i.e., PS2 is much more cytotoxic than PS1.

**Table 7:** χ, η, *S*, ω, and μ Properties Were Calculated for PS1 and PS2

S. no.	compd	energy gap (eV)	hardness (η)	softness (*S*)	electronegativity (χ)	electrophilicity index (ω)	chemical potential (μ)
1	PS1	0.1140	0.11397	4.3871	0.1418	0.0881	–0.1418
2	PS2	0.1139	0.0569	8.7873	0.1410	0.1747	–0.1410

## 4 Results Implication

Based on this report,^61^ the understanding of the integrated interaction of PYD with BSA under different environmental conditions is still very limited. To overcome this obstacle, we performed detailed studies using computational methods and supported them with experimental studies, while the MD study understands the dynamic behavior of proteins due to binding to ligands/drugs at different time intervals.^28^ Moreover, the combined approach of molecular mechanics with the continuum solvent approach of MMPBSA is useful to evaluate the binding energies of protein–ligand/drug complexes.^29^ Therefore, the results of these MD simulations play a key role in the methodological improvement of drug discovery and development. Moreover, these cytotoxic studies reveal the toxic potency of each compound. Thus, our detailed studies can be useful in solving drug application problems. In addition, we plan to study the interaction of PYD with human serum albumin to determine the effect of the compounds in the human body. We believe that the whole study can be useful to investigate the antibacterial effect of PS1 and PS2 on human and animal health in the future.

## 5 Conclusion

In the present work, we addressed the binding affinity of compounds PS1 and PS2 to the intrinsically fluorescent plasma protein of BSA, which was verified by various physicochemical analyses and computational methods. Our experimental data suggest that the interaction of BSA with the compounds PS follows the static quenching mechanism. The binding constants extracted from Stern–Volmer plots showed that BSA has a higher binding affinity to PS2 than to PS1, with a larger dominant hydrophobic binding force, which corresponds exactly to the usual properties of binding proteins such as albumins. The chemical potential of both compounds is almost identical, suggesting that both compounds trigger a similar mechanism in the organism. Molecular docking studies were used to further decipher the binding pathway and to investigate the interaction of compounds PS1 and PS2 with the BSA protein (BSA-PS1 and BSA-PS2 complexes). The docking studies showed a similar trend to the experimental data, and the same binding mode of BSA with the compounds to fluorescent tryptophan was observed. Molecular dynamics was used to observe the conformational alignment of the two compounds within the BSA protein. The binding energy values derived from the computational simulation were −138.50 and −99.30 kJ/mol for PS2 and PS1, respectively. These results indicate that PS2 has a higher affinity than PS1. The calculated free energy values of BSA-PS2 were more stable than those of BSA-PS1. The energy decomposition analysis shows that the electrostatic interaction plays an important role in stabilizing the binding mode of BSA-PS2. In contrast, the van der Waals interactions largely contribute to the stabilization of the binding site of BSA-PS1. The experimental values are in the micromolar range of the binding affinity of PS1 and PS2 for BSA, such as 7.39 [*K*_b_ × 10^5^ (M^–1^)] and 7.81 [*K*_b_ × 10^5^ (M^–1^)] at 298 K, indicating that the binding strength is not weak. However, they are on a different order of magnitude than the predicted values but correlate well with the experimental data. From the calculated binding energy values, PS2 is more active than PS1, and the FMO orbital transitions helped to analyze the alignment of the HOMO–LUMO orbitals of the two compounds, which follow similar paths as π → π* and n → π* of HOMO–LUMO and HOMO–1 – LUMO+1. Finally, cytotoxicity studies determined the comparative cytotoxic effect (PS2 > PS1) of these compounds. The results obtained with the MD simulation provide useful insights into the mechanism of binding of compounds to BSA at the molecular level, which will be useful in the future to assess the important factors for identifying the different mechanisms of their antibiotic action.

## Supporting Information Available

Pictorial representation of FMO view for both compounds PS1 and PS2, binding modes from MD trajectories with different time scales of two compounds interacting with BSA protein, and calculated RMSD and RMS fluctuation curve for both compounds (PDF)

## Abbreviations

PYD: pyrene derivatives
BSA: bovine serum albumin
PS1: *N*′-pyren-1-ylmethylene-hydrazinecarbodithioic acid methyl ester
PS2: *N*′-pyren-1-ylmethylene-hydrazinecarbodithioic acid benzyl ester
MDS: molecular dynamics simulation
