# Supplementary material for: Integrated Approach to Interaction Studies of Pyrene Derivatives with Bovine Serum Albumin: Insights from Theory and Experiment
Source: J Phys Chem B. 2022 May 18;126(21):3831–43. doi: 10.1021/acs.jpcb.2c00778 (PMC9169062; doi:10.1021/acs.jpcb.2c00778)
Supplement: Supplementary file 1 — jp2c00778_si_001.pdf [file jp2c00778_si_001.pdf]

Supporting information

An integrated approach to interaction studies of pyrene derivatives with bovine serum albumin: insights from theory and experiment

Selvaraj Sengottiyan[1], Kakoli Malakar[2], Arunkumar Kathiravan[3], Marappan Velusamy[2], Alicja Mikolajczyk[1], Tomasz Puzyn[1]*

[1]Laboratory of Environmental chemoinformatics, Faculty of Chemistry, University of Gdansk, Wita Stwosza 63, Gdansk, 80–308 Poland

[2]Department of Chemistry, North Eastern Hill University, Shillong 793 022, Meghalaya, India

[3]Department of Chemistry, Vel Tech Rangarajan Dr Sagunthala R & D Institute of Science and Technology, Avadi, Chennai-600 062, Tamil Nadu, India.

Corresponding author

*E-mail: tomasz.puzyn@ug.edu.pl (Prof. dr. hab. Tomasz Puzyn).

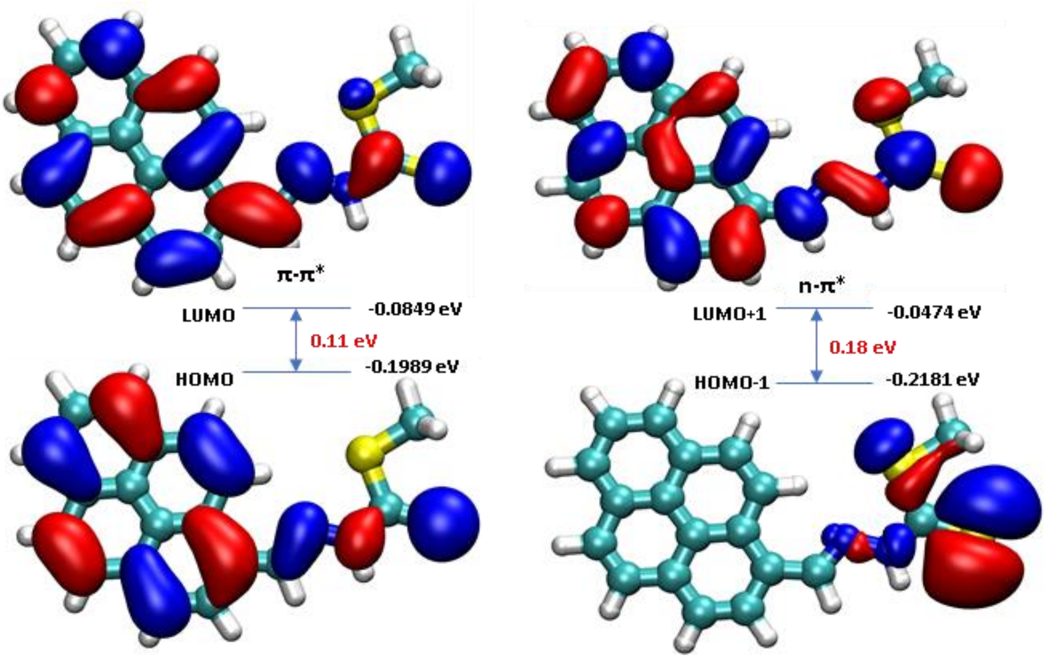

Figure S1: FMO orbitals for the PS1 compound.

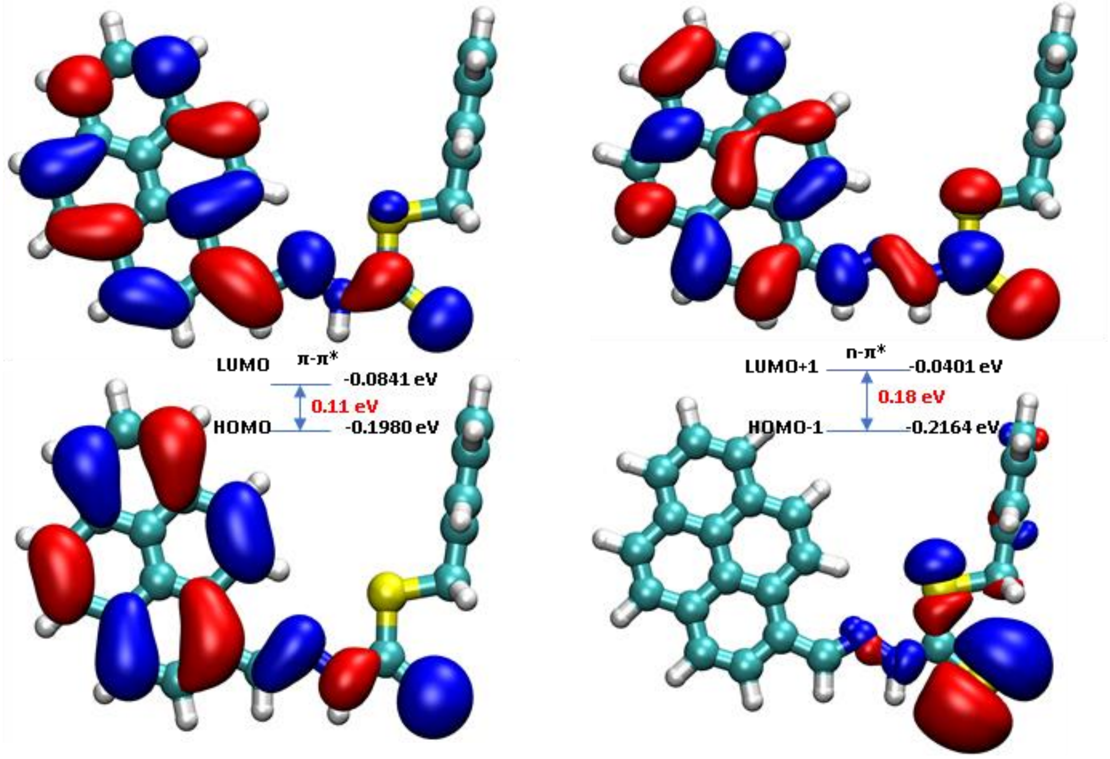

Figure S2: FMO orbitals for PS2 compound.

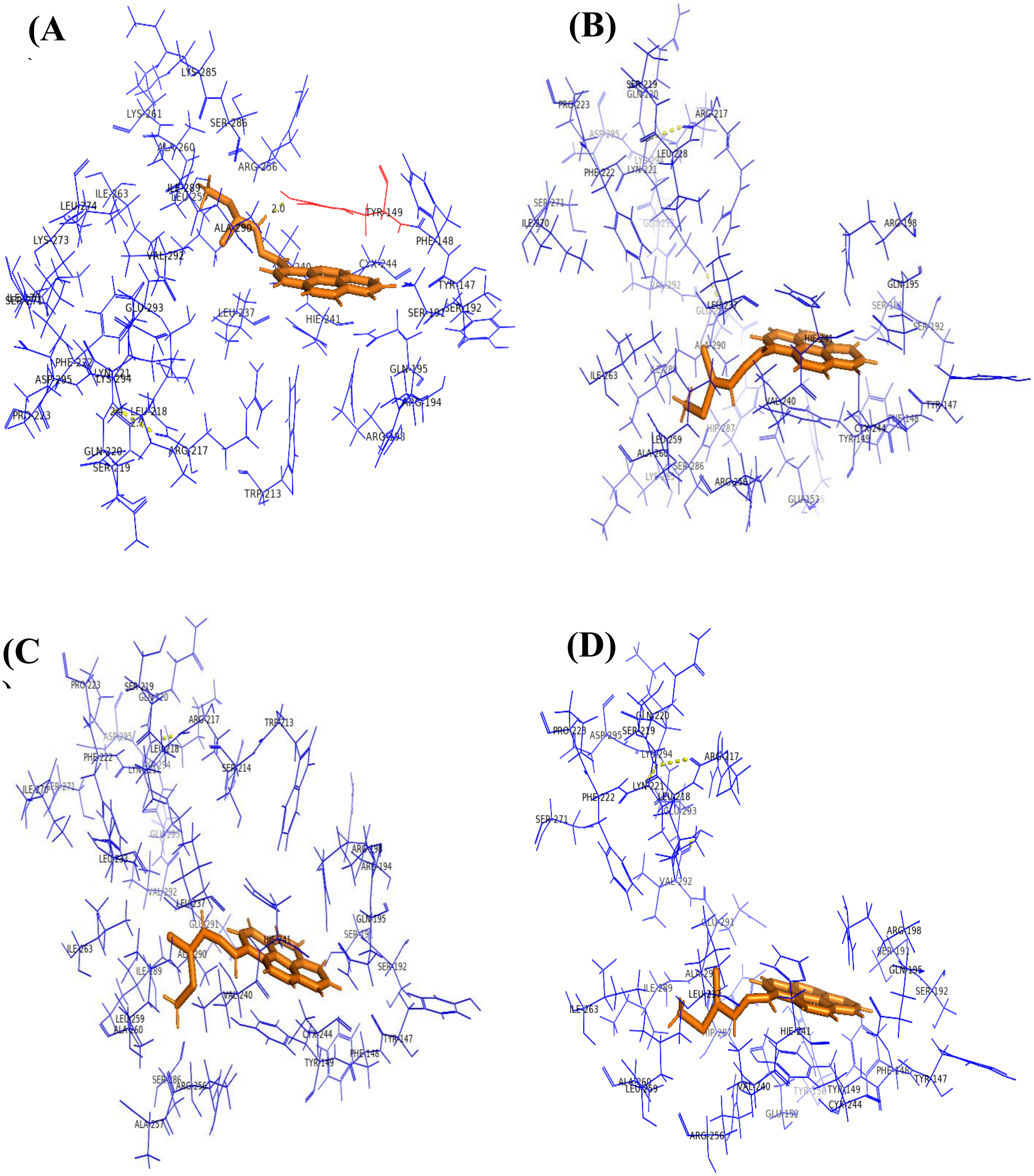

Figure S3. Binding modes of PS1 with BSA at different time scales (A) 15ns (B) 20ns (C) 30ns (D) 40 ns.

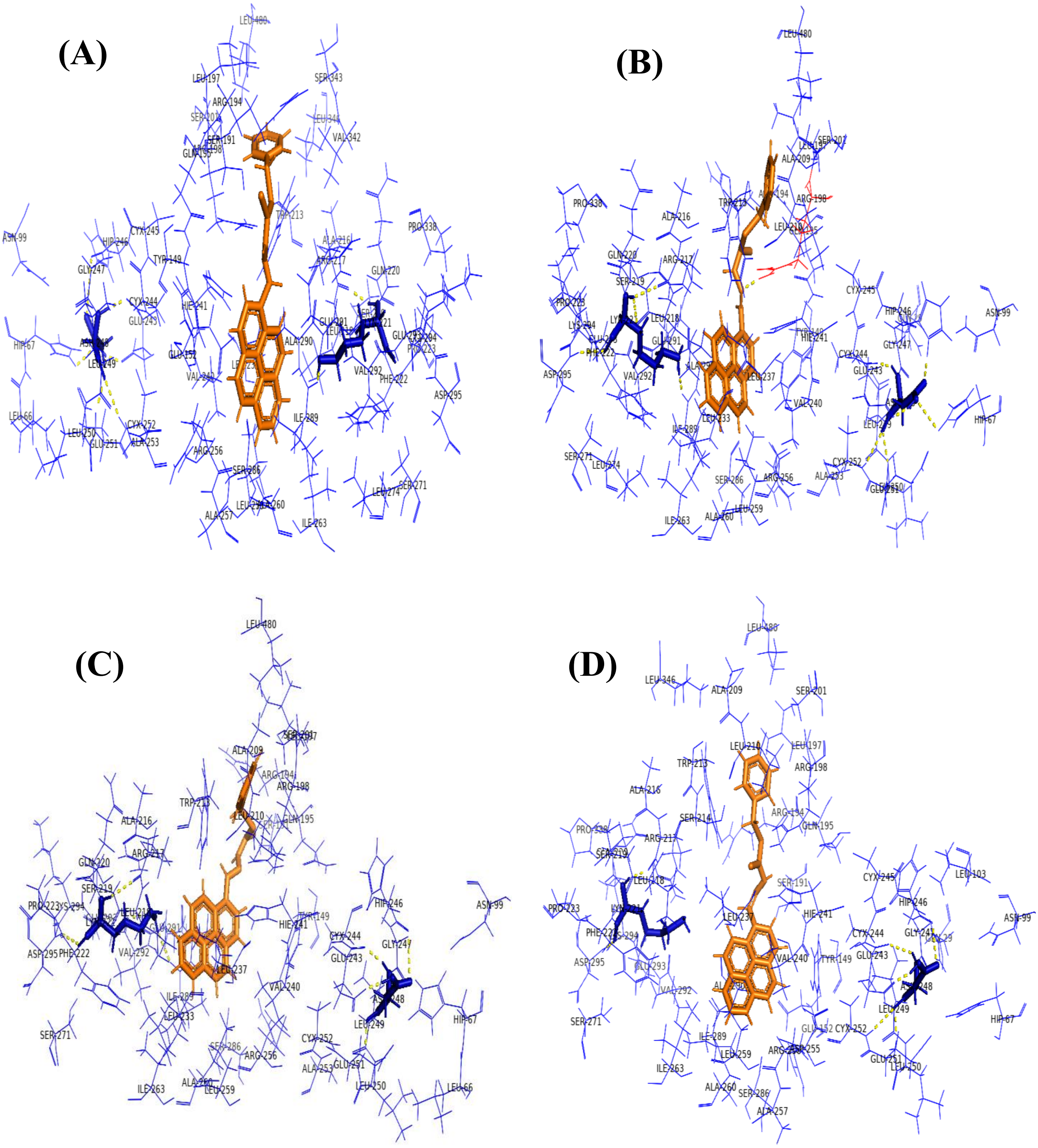

Figure S4. Binding modes of PS2 with BSA at different time scales (A) 15ns (B) 20ns (C) 30ns (D) 40 ns.

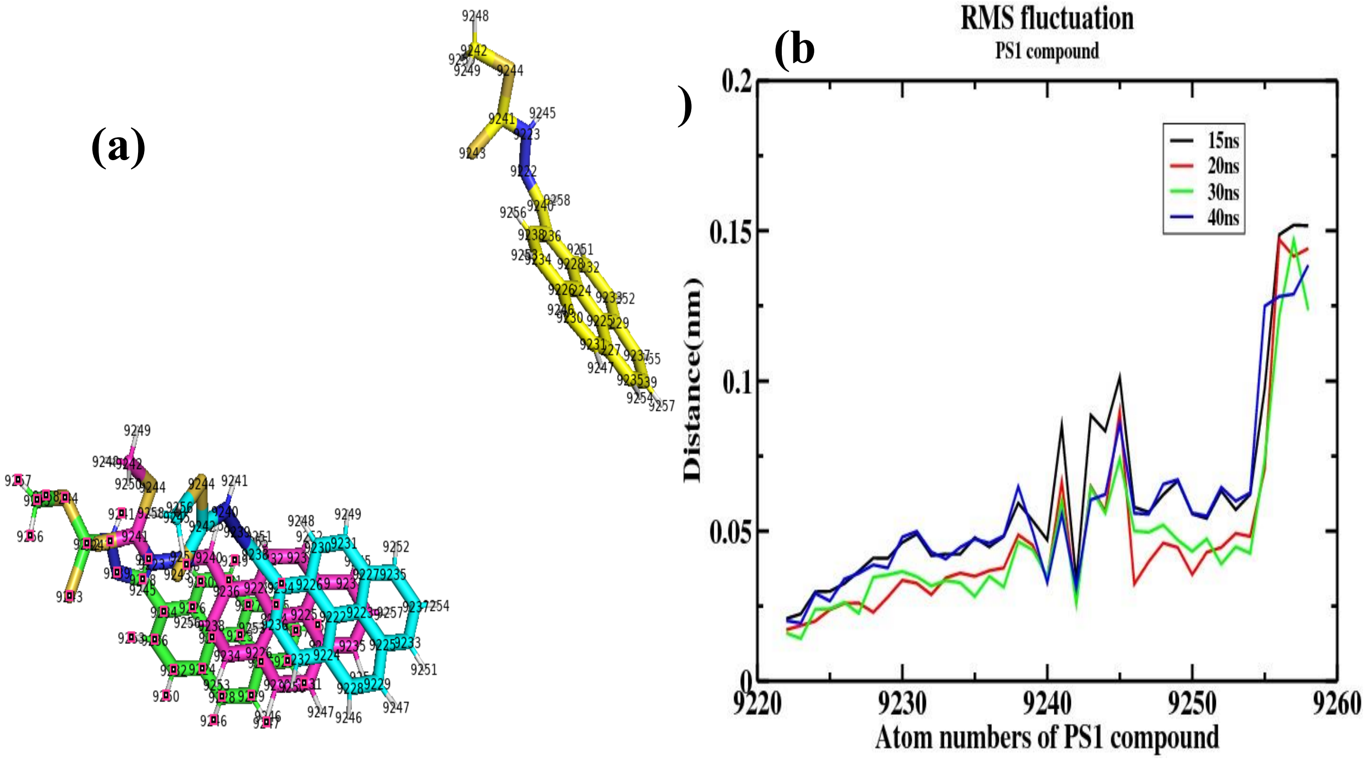

Figure. S5. (a) The overlapping view of the four snapshots of the PS1 compound at different time intervals such as 15, 20, 30, and 40 ns. (b) The root means square fluctuation (RMSF) of each atom of the PS1 compound during different time intervals at 15, 20, 30, and 40 ns.

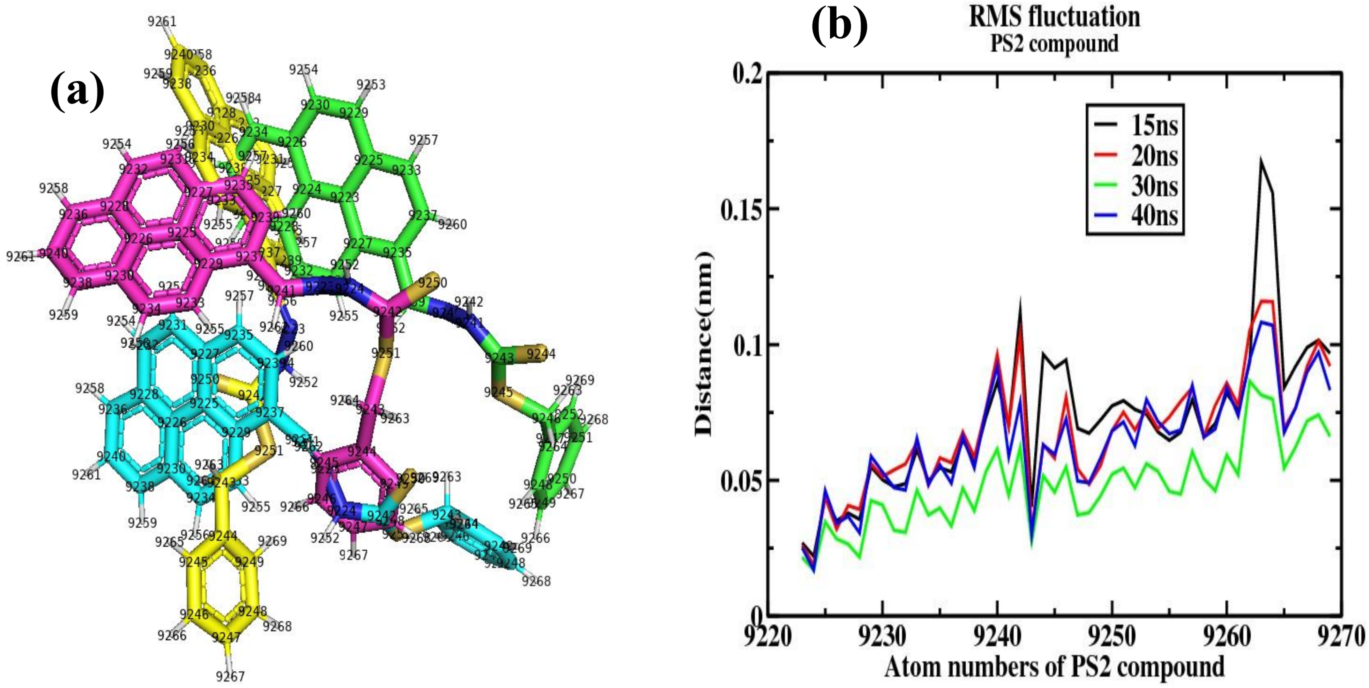

Figure. S6. (a) The overlapping view of the four snapshots of the PS1 compound at different time intervals such as 15, 20, 30, and 40 ns.

(b) The root means square fluctuation (RMSF) of each atom of the PS1 compound during different time intervals at 15, 20, 30, and 40 ns.

Table S1: Conformational alignment of PS1 and PS2 in each of four snapshots extracted from the MD trajectory using the Gromacs tools.

Time (ns)[a]	RMSD of PS1(Å)[a,b]	RMSD of PS2(Å)[a,b]	Interacting residues of complex BSA-PS1	Interacting residues of complex BSA-PS2
**15**	0.09	0.12	TYR149 - H-bond	No residues involved in H-bond
**20**	0.08	0.12	No residues involved in H-bond	ARG198 - H-bond
**30**	0	0	No residues involved in H-bond	No residues involved in H-bond
**40**	0.10	0.23	No residues involved in H-bond	No residues involved in H-bond

[a]The obtained average of frame numbers such as 1500, 2000, 3000, and 4000 concerning 15, 20, 30, and 40 ns from the entire MD trajectory.

[b]The RMSD represents the relative position shifts concerning the reference structure of 30 ns, the RMSD values calculated according to the no fit RMSD scheme, which includes the translational and rotational motions of PS1 and PS2, respectively.
